# Optic Disc Pseudoduplication Following Focal Laser Application for Non-center Involved Clinically Significant Diabetic Macular Edema: A Case Report

**DOI:** 10.7759/cureus.102081

**Published:** 2026-01-22

**Authors:** Asli Perente, Doukas Dardabounis, Aikaterini Giannoukaki, Tryfon Rotsos, Georgios Labiris

**Affiliations:** 1 Department of Ophthalmology, East Lancashire Hospitals NHS Trust, Blackburn, GBR; 2 Department of Ophthalmology, University Hospital of Alexandroupolis, Alexandroupolis, GRC; 3 First Department of Ophthalmology, G. Gennimatas General Hospital, University of Athens, Athens, GRC

**Keywords:** choroidal neovascular membrane, diabetic macular edema, focal laser treatment, optic disc pseudo-doubling, optic disc pseudoduplication

## Abstract

Optic disc pseudoduplication, or pseudo-doubling, is a rare clinical condition that refers to the presence of an optic disc-like lesion located adjacent to the true optic nerve head, creating the impression of a duplicated disc. The vast majority of reported cases are associated with optic disc or chorioretinal coloboma. In this article, we present a rare example of pseudo-disc formation that developed secondary to a laser-induced choroidal neovascular membrane, highlighting its unusual appearance.

## Introduction

Optic disc duplication is a rare clinical entity that can be either true doubling or pseudo-doubling. The diagnosis of true doubling of the optic nerve head is considered to be extremely rare in humans but more common in lower vertebrates. It refers to the separation of the optic nerve into two fasiculi and the presence of a dual system of retinal vessels [1]. Τhe term pseudoduplication is used when there is an optic disc-like lesion adjacent to the true optic disc with radiating blood vessels [2]. In this condition, although a double blind spot on visual field testing can be detected, there is neither an independent vascular supply nor an ultrasound or CT image of the second optic nerve [3]. Regarding the associated vasculature observed in these cases, researchers have pointed out that these are normal retinal vessels passing through the lesion, thus giving the pseudo appearance of independent vascularization [4]. Several disorders can mimic the appearance of optic disc, including optic disc coloboma, peripapillary chorioretinal coloboma, and degenerative myopia scarring [5-7]. We report a case of pseudo-disc-like chorioretinal defect in a patient with non-proliferative diabetic retinopathy following focal laser treatment for clinically significant macular edema.

## Case presentation

A 66-year-old female with a history of non-proliferative diabetic retinopathy presented to our clinic for a routine follow-up examination. Her ophthalmic history was notable for prior argon green laser photocoagulation for non-center-involved clinically significant macular edema in both eyes, performed 13 years earlier (power, 150 mW; spot size, 100 μm; exposure time, 100 ms).

At presentation, best-corrected visual acuity was 0.2 logMAR in the right eye (OD) and 0.4 logMAR in the left eye (OS). Intraocular pressure measured 14 mmHg OD and 16 mmHg OS. Anterior-segment examination revealed age-related cataracts in both eyes, with a deep and quiet anterior chamber.

**Figure 1:**
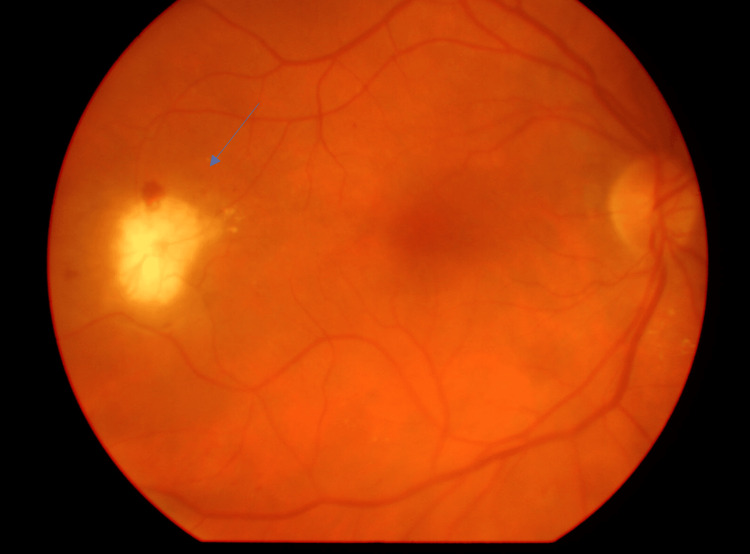
Color fundus photograph of the right eye demonstrating the optic disc-like lesion temporal to the fovea (blue arrow).

Dilated fundus examination of the right eye revealed a newly identified, well-circumscribed optic disc-like lesion located temporally to the fovea, with visible superficial vessels resembling those of the optic nerve head (Figure 1). This lesion had not been present at the patient’s previous examination six months earlier. The left eye remained stable compared to prior visits.

**Figure 2:**
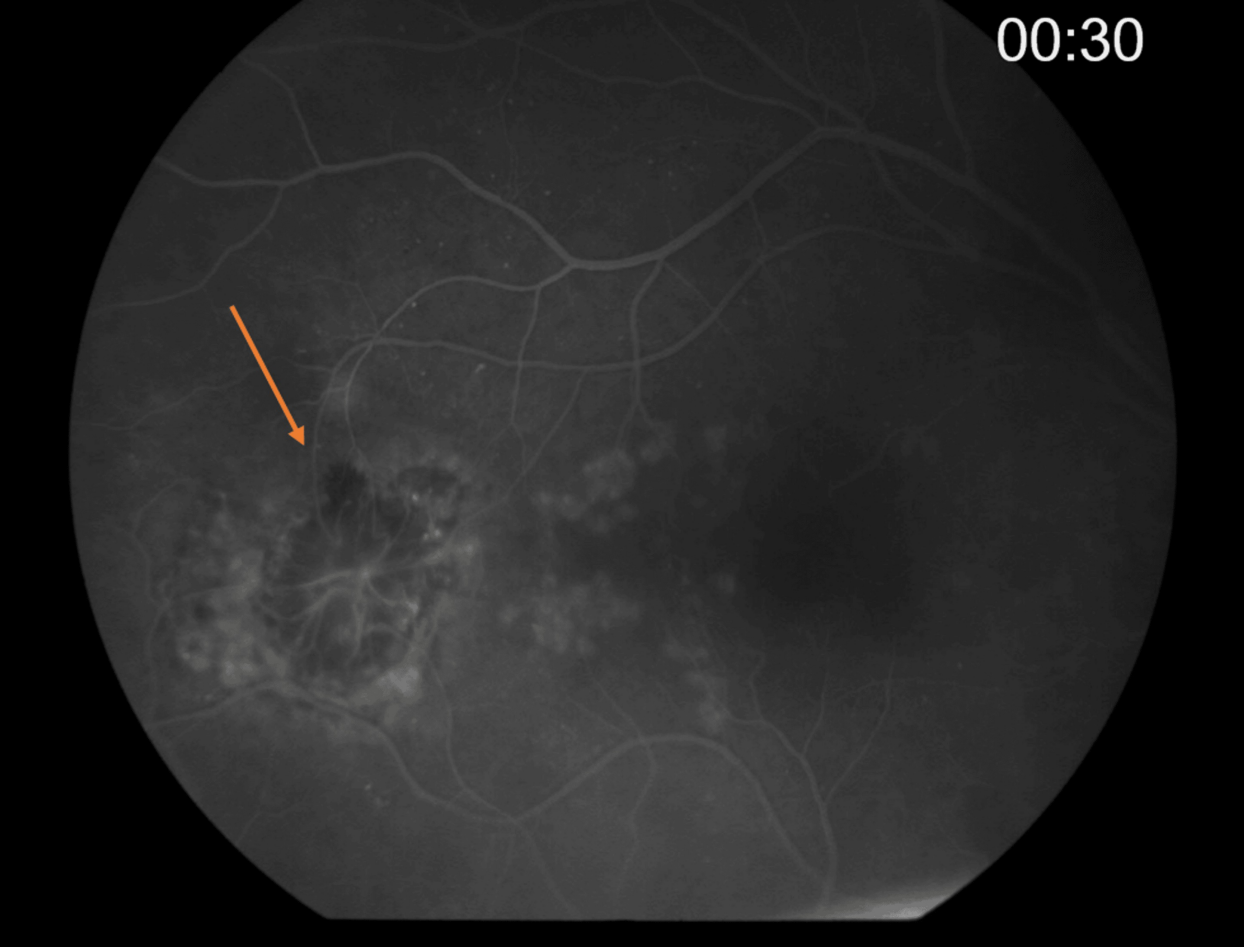
Fluorescein angiography of the right eye showing early hypofluorescence of the lesion (orange arrow).

**Figure 3:**
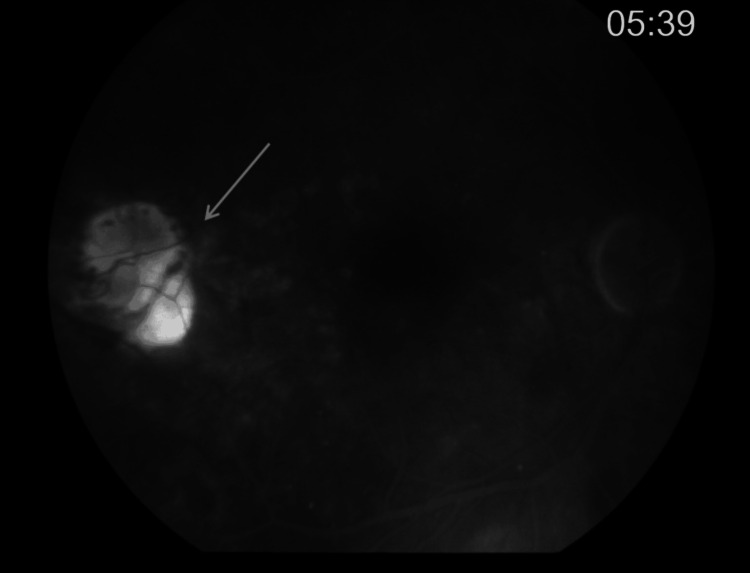
Late-phase fluorescein angiography of the right eye showing late hyperfluorescence of the optic-disc like lesion (grey arrow).

**Figure 4:**
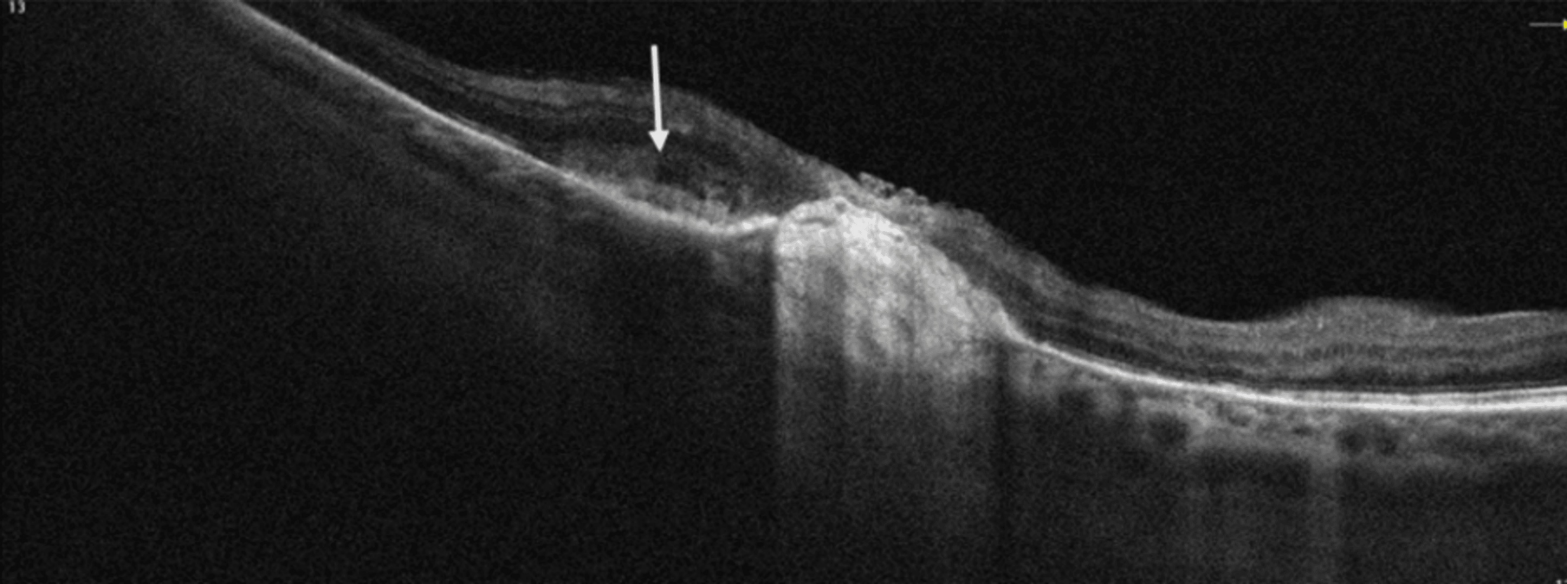
Optical coherence tomography of the right eye showing the presence of subretinal and intraretinal fluid.

**Figure 5:**
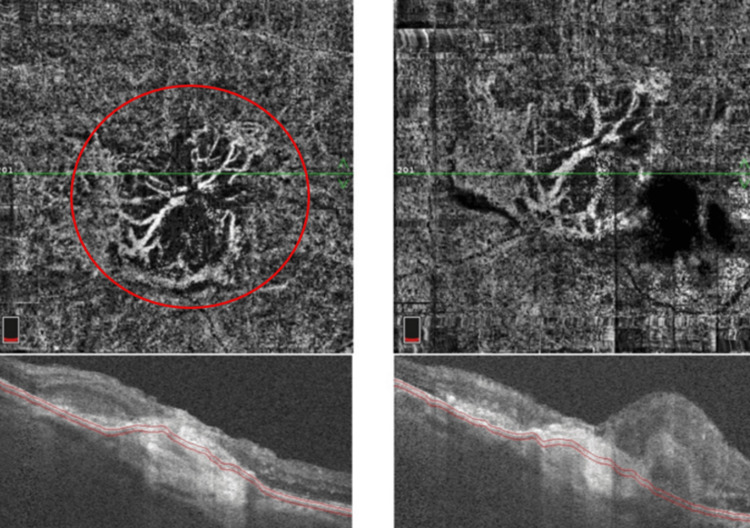
Optical coherence tomography angiography of the right eye depicting the neovascular network above the level of the retinal pigment epithelium.

Further multimodal imaging was performed to characterize the lesion. Fluorescein angiography demonstrated early hypofluorescence with late staining, and a central vascular network resembling normal optic disc vasculature (Figures 2, 3). Optical coherence tomography revealed the presence of both subretinal and intraretinal fluid adjacent to the lesion (Figure 4). Optical coherence tomography angiography showed a neovascular network located in the subretinal space, above the level of the retinal pigment epithelium (Figure 5). These findings were consistent with the presence of a choroidal neovascular membrane.

Given the extrafoveal location of the lesion, minimal exudative activity, and preserved visual acuity, a conservative approach with close observation was chosen. Serial optical coherence tomography imaging demonstrated a gradual reduction in exudative changes, with complete resolution of both subretinal and intraretinal fluid at 12 months of follow-up.

Considering the characteristic optic disc-like appearance, the imaging features of subretinal neovascularization, and the lesion’s location at the margins of previous focal laser scars, this presentation was interpreted as optic disc pseudoduplication secondary to choroidal neovascular membrane formation. The lesion was considered a possible late sequela of prior focal laser photocoagulation rather than a definitive causal complication.

## Discussion

The majority of optic disc pseudoduplication cases reported in the literature are attributed to choroidal coloboma, resulting from incomplete closure of the fetal fissure [8]. These lesions are typically located inferior to the normal optic nerve head and are characterized by the absence of the choroid, retinal pigment epithelium, and retina in the affected area [9]. Islam et al. reported that all 11 cases in their non-comparative retrospective series represented chorioretinal coloboma and emphasized the presence of bridging retinal vessels between the true optic disc and the disc-like lesion as a characteristic clinical finding [1]. Cellini et al. further demonstrated that such vessels may, in some cases, originate from the choroidal circulation [9]. Bloom et al. described an unusual case of optic disc pseudoduplication secondary to a peripheral chorioretinal scar in a patient with proliferative diabetic retinopathy, suggesting that the vessels arising from the pseudo-disc were purely choroidal and represented diabetic neovascularization [4].

In the present case, the optic disc-like lesion was identified as a choroidal neovascular membrane, which was considered a possible late sequela of prior focal laser photocoagulation. Secondary choroidal neovascularization is a recognized complication of laser photocoagulation performed for various retinal conditions, including central serous chorioretinopathy, diabetic macular edema, and branch retinal vein occlusion [10-12]. Regardless of the underlying indication, the proposed pathophysiologic mechanism is similar: laser-induced disruption of Bruch’s membrane may trigger proliferation of abnormal vessels from the choriocapillaris [13]. Although choroidal neovascularization most commonly develops within weeks to months following laser treatment, delayed presentations occurring several years later have also been reported [11]. In our patient, the membrane developed 13 years after laser application, supporting the possibility of a very late manifestation.

Intravitreal anti-vascular endothelial growth factor therapy currently represents the gold standard first-line treatment for choroidal neovascularization across most etiologies. Photodynamic therapy is now largely reserved for selected cases or historical indications and is used far less frequently in contemporary practice. In the present case, although the lesion demonstrated imaging features of activity at diagnosis, the associated intraretinal and subretinal fluid was extrafoveal and did not affect visual acuity. In such scenarios, conservative management with close observation has been described as an acceptable strategy in selected patients with extrafoveal or minimally active choroidal neovascularization and preserved central vision [14]. Accordingly, the patient was monitored without immediate intervention, and spontaneous resolution of exudative changes was observed within 12 months.

Collectively, the optic disc-like clinical appearance, multimodal imaging findings consistent with subretinal neovascularization, and the lesion’s spatial relationship to prior focal laser scars support the diagnosis of optic disc pseudoduplication secondary to choroidal neovascular membrane formation.

## Conclusions

This case highlights a rare instance of optic disc pseudoduplication-like chorioretinal defect caused by choroidal neovascularization as a late complication of focal laser photocoagulation for diabetic macular edema. It underscores the importance of long-term follow-up in patients who have undergone retinal laser treatment, as complications may arise years later. To our knowledge, this is the first reported case of optic disc pseudo-doubling following focal laser treatment for diabetic macular edema.
